# Protocadherin-7 Regulates Monocyte Migration Through Regulation of Small GTPase RhoA and Rac1

**DOI:** 10.3390/ijms26020572

**Published:** 2025-01-11

**Authors:** Hyunsoo Kim, Noriko Takegahara, Yongwon Choi

**Affiliations:** Department of Pathology and Laboratory Medicine, University of Pennsylvania Perelman School of Medicine, Philadelphia, PA 19104, USA; hyunsoo3@pennmedicine.upenn.edu (H.K.); tnoriko@pennmedicine.upenn.edu (N.T.)

**Keywords:** monocyte migration, Pcdh7, GSK3β, PP2A, RhoA, Rac1

## Abstract

Protocadherin-7 (Pcdh7) is a member of the non-clustered protocadherin δ1 subgroup within the cadherin superfamily. Pcdh7 has been shown to control osteoclast differentiation via the protein phosphatase 2A (PP2A)–glycogen synthase kinase-3β (GSK3β)–small GTPase signaling axis. As protocadherins serve multiple biological functions, a deeper understanding of Pcdh7’s biological features is valuable. Using an in vitro mouse monocyte cell culture system, we demonstrate that Pcdh7 plays a role in regulating monocyte migration by modulating the small GTPases RhoA and Rac1. Pcdh7-deficient (*Pcdh7^−/−^*) bone marrow-derived monocytes exhibited impaired migration along with the reduced activation of RhoA and Rac1. This impaired migration was rescued by transduction with constitutively active forms of RhoA and Rac1. Treatment with the PP2A-specific activator DT-061 enhanced cell migration, whereas treatment with the GSK3β-specific inhibitor AR-A014418 inhibited migration in wild-type monocytes. In contrast, treatment with DT-061 failed to restore the impaired migration in *Pcdh7^−/−^* monocytes. These findings suggest the involvement of PP2A and GSK3β in monocyte migration, although the forced activation of PP2A alone is insufficient to restore impaired migration in *Pcdh7^−/−^* monocytes. Taken together, these results indicate that Pcdh7 regulates monocyte migration through the activation of RhoA and Rac1. Given the pivotal role of cell migration in both physiological and pathological processes, our findings provide a foundation for future research into therapeutic strategies targeting Pcdh7-regulated migration.

## 1. Introduction

Protocadherins (Pcdhs), a distinct subfamily within the broader cadherin superfamily, play crucial roles in various processes, such as cell adhesion [1]. To date, over 70 different Pcdh genes have been identified [2]. These proteins are primarily expressed during the early developmental stages of the nervous system, particularly in the brain and heart, with minimal expression in other tissues such as the stomach, thyroid, spinal cord, and placenta [3]. Structurally, Pcdhs differ from classical cadherins in that they possess more than five extracellular cadherin domains and lack catenin-binding sites in their cytoplasmic regions. While classical cadherins mediate cell–cell adhesion through homophilic interactions, Pcdhs appear to have more varied functions, such as mediating cell–cell adhesion or regulating other molecules [4]. Pcdhs are classified into two groups: clustered and non-clustered Pcdhs. Non-clustered Pcdhs have been shown to perform multiple biological functions under both physiological and pathological conditions across various tissues [5,6,7,8,9,10,11]. Previously, we demonstrated that Pcdh7, a member of the non-clustered Pcdhs, regulated osteoclast differentiation by controlling PP2A-mediated dephosphorylation of GSK3β [12,13,14]. PP2A is a multi-subunit enzyme composed of three subunits—the catalytic (C), scaffolding (A), and regulatory (B) subunits—which together form an active holoenzyme complex [15]. GSK3β is a serine/threonine protein kinase regulated by inhibitory phosphorylation at Ser9, and PP2A dephosphorylates it [16,17,18]. This dephosphorylation is required for the activation of small GTPases, including RhoA, during osteoclast differentiation [14]. Our findings underscore the pivotal role of this pathway in proper osteoclast differentiation.

Cell migration is fundamental to various biological and pathological processes, including embryonic development, wound healing, angiogenesis, inflammation, tumor invasion, and metastasis [19]. Cell movement is primarily driven by the dynamic reorganization of the cytoskeleton, which enables protrusion at the cell front and retraction at the back [20]. This process is mainly regulated by small GTPases of the Rho family, particularly RhoA, Rac, and Cdc42 [21,22,23]. The main cytoskeletal components responsible for generating the migratory force are F-actin and myosin II motor proteins, collectively referred to as actomyosin. Small GTPases critically regulate cellular actomyosin dynamics through their immediate downstream effector kinases, including Rho-associated coiled-coil-containing protein kinase (ROCK) and p21-activated kinase (PAK) [24]. Among these, the importance of RhoA is evident from the observation that pharmacological inhibition or macrophage-specific RhoA deletion attenuates cell migration, mitigating macrophage-mediated chronic rejection and neuronal disorders [25,26,27]. Rac plays a key role by promoting actin polymerization and the assembly of integrin adhesion complexes, both of which are essential for membrane protrusion, cell spreading, and migration [28]. In addition to small GTPases, other signaling molecules have been reported to be involved in regulating this process, including phosphatases such as the phosphatase and tensin homolog (PTEN), Src homology 2-containing protein tyrosine phosphatase-2 (SHP-2), protein tyrosine phosphatase 1B (PTP1B), and PP2A [29]. Notably, PP2A has been shown to regulate cell migration [30,31,32], and mutations in genes encoding PP2A subunits have been associated with several pathological conditions, including myopathy and bone cell-mediated diseases [33,34,35]. These findings highlight the critical roles of these molecules in cell migration and position them as potential therapeutic targets.

Given Pcdh7’s role in regulating PP2A and small GTPase activation, along with the importance of these signaling molecules in cell migration, it is plausible that Pcdh7 influences this process. Monocytes, a type of myeloid lineage cell and precursor of osteoclasts, possess notable migratory capabilities. In this study, using bone marrow-derived monocytes, we demonstrated that Pcdh7 contributes to cell migration through the activation of RhoA and Rac1. Pcdh7 deficiency resulted in impaired monocyte migration, accompanied by the reduced activation of RhoA and Rac1, which was rescued by transduction with constitutively active forms of RhoA and Rac1. Enhancing PP2A activity with the specific activator DT-061 improved cell migration, while inhibiting GSK3β activity with the specific inhibitor AR-A014418 reduced cell migration in Pcdh7-intact cells. However, treatment with DT-061 was insufficient to restore the impaired migration in Pcdh7-deficient cells. These findings demonstrate that Pcdh7 is a critical regulator of monocyte motility through the regulation of small GTPases.

## 2. Results

### 2.1. Pcdh7 Deficiency Results in Monocytes with Impaired Motility 

**Figure 1:**
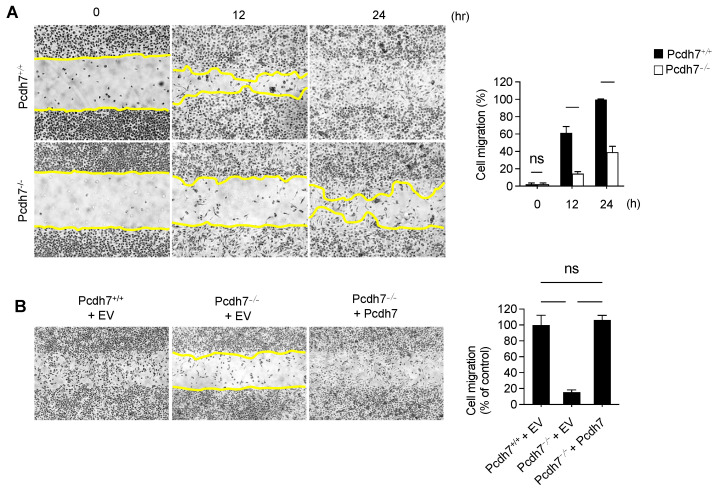
Pcdh7 is involved in monocyte migration. (**A**) The cell migration of *Pcdh7^+/+^* and *Pcdh7^−/−^* BMMs. *Pcdh7^+/+^* and *Pcdh7^−/−^* BMMs were cultured with macrophage colony-stimulating factor (M-CSF) (60 ng/mL) for the indicated times, and the migrated area was measured. The yellow lines mark the area lacking cells. (**B**) Monocyte migration rescued by the retroviral transduction of Pcdh7 in *Pcdh7^−/−^* cells. *Pcdh7^+/+^* and *Pcdh7^−/−^* BMMs were retrovirally transduced with either the empty vector (EV) or Pcdh7 expression vector, followed by culture with M-CSF. The percentage of the migration area is shown. Data are presented as mean ± S.D. ns: not significant.

First, we sought to identify the motility of monocytes in the presence or absence of Pcdh7 using an in vitro monolayer scratch assay with bone marrow-derived monocytes (BMMs) from wild-type mice (*Pcdh7^+/+^*) and mice lacking Pcdh7 (*Pcdh7^−/−^*). Control *Pcdh7^+/+^* monocytes exhibited migration that filled the cell-denuded gap within 24 h. In contrast, *Pcdh7^−/−^* cells showed reduced migration into the gap compared to wild-type (*Pcdh7^+/+^*) cells (Figure 1A). The retroviral transduction of *Pcdh7^−/−^* BMMs with full-length Pcdh7 rescued the impaired cell migration (Figure 1B), suggesting that the impaired motility observed in *Pcdh7^−/−^* monocytes is attributed to the absence of the Pcdh7 gene.

### 2.2. Pcdh7-Mediated Activation of RhoA and Rac1 Is Required for Monocyte Motility

**Figure 2:**
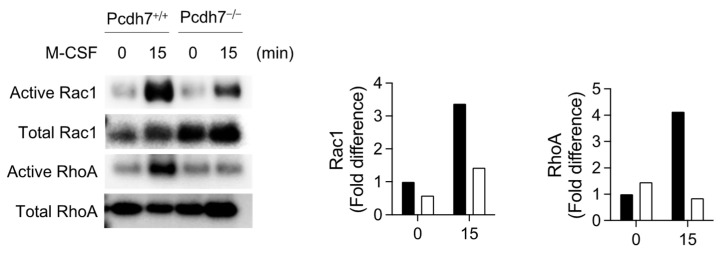
Pcdh7 is required for the activation of RhoA and Rac1 in monocytes. *Pcdh7^+/+^* and *Pcdh7^−/−^* BMMs were stimulated with M-CSF (60 ng/mL) for 15 min, and the activated forms of RhoA and Rac1 were detected using a pull-down assay. Representative Western blotting results are shown (**left**). The RhoA and Rac1 activation levels were quantified (**right**).

We have previously demonstrated the involvement of the small GTPases RhoA and Rac1 in Pcdh7-mediated signaling in osteoclasts [14]. Given the critical roles of RhoA and Rac1 in cell motility, we aimed to determine the activation status of these molecules in *Pcdh7^−/−^* monocytes. BMMs from *Pcdh7^+/+^* and *Pcdh7^−/−^* mice were stimulated with M-CSF, and the activation of RhoA and Rac1 was assessed using a pull-down assay. M-CSF stimulation activated RhoA and Rac1 in *Pcdh7^+/+^* cells (Figure 2). In contrast, the activation of RhoA and Rac1 was dramatically reduced in *Pcdh7^−/−^* cells (Figure 2). These results suggest that Pcdh7 is required for sufficient activation of RhoA and Rac1 in M-CSF-cultured BMMs.

**Figure 3:**
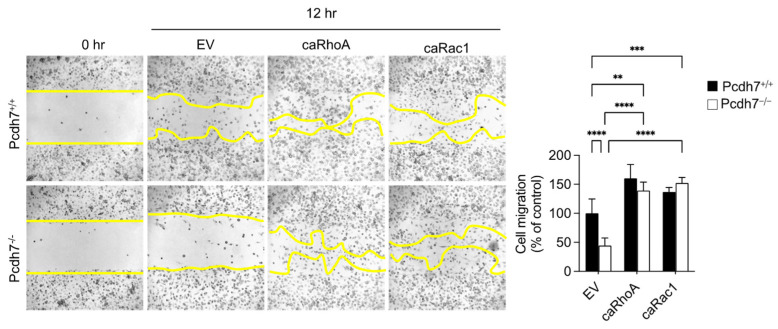
Constitutively active forms of RhoA and Rac1 rescue the impaired migration of *Pcdh7^−/−^* BMMs. *Pcdh7^+/+^* and *Pcdh7^−/−^* BMMs were retrovirally transduced with the indicated vectors, followed by culture with M-CSF for the indicated time. The cell-migrated area was measured. The yellow lines mark the area lacking cells. The percentage of the migrated area is shown. Data are presented as mean ± S.D. *p* ** < 0.01, *p* *** < 0.005, and *p* **** < 0.0001.

We then investigated whether the impaired activation of RhoA and Rac1 is the key factor responsible for the reduced motility observed in *Pcdh7^−/−^* cells. *Pcdh7^+/+^* and *Pcdh7^−/−^* cells were retrovirally transduced with constitutively active forms of RhoA (caRhoA) and Rac1 (caRac1), followed by an assessment of their motility using a scratch assay. The expressions of caRhoA and caRac1 rescued the impaired migration of *Pcdh7^−/−^* cells and enhanced the migration of *Pcdh7^+/+^* cells (Figure 3). These results suggest that the Pcdh7-mediated activation of RhoA and Rac1 is essential for the motility of BMMs.

### 2.3. Involvement of PP2A and GSK3β in Monocyte Migration

**Figure 4:**
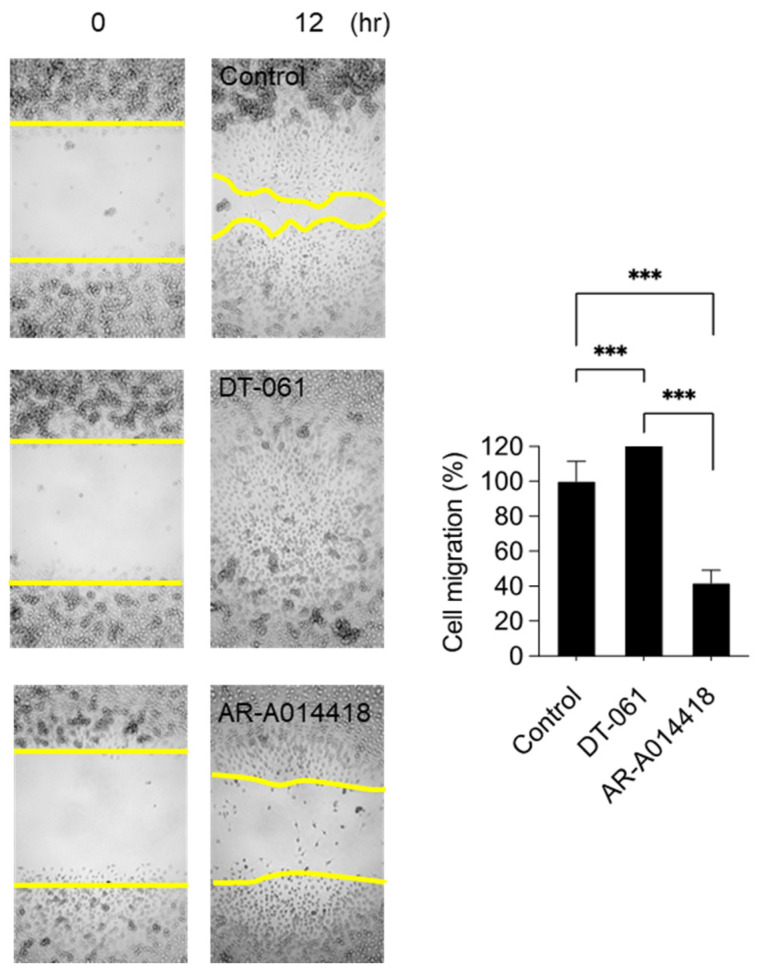
The modulation of PP2A or GSK3β activities affects BMM migration. *Pcdh7^+/+^* BMMs were cultured with M-CSF (60 ng/mL) for the indicated times in the presence of the PP2A-specific activator DT-061 (10 μM) or GSK3β-specific inhibitor AR-A014418 (10 μM). The cell-migrated area was measured. The yellow lines mark the area lacking cells. The percentage of the migrated area is shown. Data are presented as mean ± S.D. *p* *** < 0.005.

**Figure 5:**
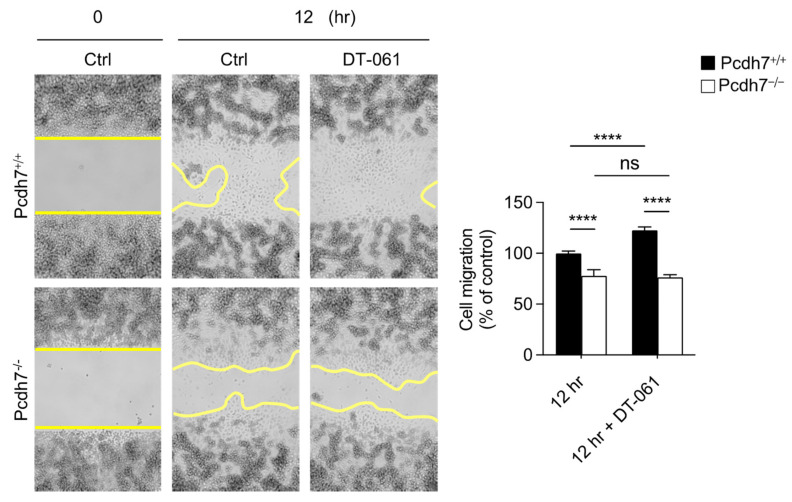
The forced activation of PP2A does not rescue the impaired migration of *Pcdh7^−/−^* BMMs. *Pcdh7^+/+^* and *Pcdh7^−/−^* BMMs were cultured with M-CSF for the indicated times in the presence or absence of the PP2A-specific activator DT-061 (10 μM). The cell-migrated area was measured. The yellow lines mark the area lacking cells. The percentage of the migrated area is shown. Data are presented as mean ± S.D. *p* **** < 0.0001. ns: not significant.

We previously demonstrated the involvement of the Pcdh7–PP2A–GSK3β signaling axis in osteoclasts: Pcdh7 recruits PP2A, which dephosphorylates and activates GSK3β, leading to the activation of the small GTPase RhoA [14]. To gain insight into the roles of PP2A and GSK3β in monocyte motility, we employed the small compounds of DT-061, an activator of PP2A, and AR-A014418, an inhibitor of GSK3β, to modulate the activities of PP2A and GSK3β during migration. *Pcdh7^+/+^* BMMs treated with DT-061 exhibited a significant enhancement of migration compared to the control, while those treated with AR-A014418 showed a dramatic reduction in migration (Figure 4), suggesting the involvement of PP2A and GSK3β in monocyte migration. Notably, the treatment of *Pcdh7^−/−^* BMMs with DT-061 did not restore their impaired migration (Figure 5). These results suggest that the activation of PP2A alone is insufficient to rescue the impaired migration in Pcdh7-deficient cells. Taken together, our findings demonstrate the role of Pcdh7 in monocyte migration through the activation of RhoA and Rac1.

## 3. Discussion

Although we have previously demonstrated that Pcdh7 expression is drastically increased by receptor-activated NF-kB ligand (RANKL) stimulation, a substantial amount of Pcdh7 was also detected in *Pcdh7^+/+^* monocytes [12,14]. These observations suggest a potentially significant role of Pcdh7 in monocytes. In the present study, we investigated the role of the Pcdh7 in monocytes and demonstrated its requirement for monocyte migration. The impaired migration of *Pcdh7^−/−^* monocytes was completely restored by the retroviral transduction of Pcdh7, indicating a cell-intrinsic role for Pcdh7 in controlling monocyte movement. Notably, the involvement of Pcdh7 in cell migration has been reported in cancer cell lines, including PC-3 and RPE1 [36,37]. These observations suggest a crucial role for Pcdh7 in the regulation of migration under both physiological and pathological conditions.

Previously, we showed that the activation of the small GTPases RhoA and Rac1 was dramatically diminished in *Pcdh7^−/−^* osteoclasts [13]. Here, we demonstrated that the activation of RhoA and Rac1 was also reduced in *Pcdh7^−/−^* monocytes. The transduction of caRhoA and caRac1 completely restored and even enhanced the impaired migration of *Pcdh7^−/−^* monocytes. These findings suggest that Pcdh7 is required for the activation of RhoA and Rac1, underscoring its fundamental role in regulating these GTPases and its necessity for monocyte motility. Moreover, the impaired migration of *Pcdh7^−/−^* monocytes could be attributed at least partly, if not entirely, to the impaired activation of RhoA and Rac1. The well-documented importance of these GTPases and their associated signaling pathways in cell migration underscores their contribution to the Pcdh7-mediated regulation of cell migration [24,38,39,40].

We previously demonstrated that the PP2A–GSK3β signaling axis is required for the activation of RhoA downstream of Pcdh7 in osteoclasts [14]. In this study, we showed that the inhibition of GSK3β with the specific inhibitor AR-A014418 suppressed the migration of *Pcdh7^+/+^* monocytes. As AR-A014418 can abolish RhoA activation [14], and because the phosphorylation of GSK3β at Ser9—which inhibits GSK3β activity—was increased in *Pcdh7^−/−^* monocytes [14], it is plausible that (1) the inhibition of GSK3β with AR-A014418 inhibited cell migration through the suppression of small GTPases, including RhoA, and (2) the impaired migration of *Pcdh7^−/−^* monocytes could be attributed at least in part to the enhanced phosphorylation of GSK3β. PP2A contributes to the dephosphorylation of GSK3β at Ser 9 downstream of Pcdh7 [14]. We revealed that the activation of PP2A with the specific PP2A activator DT-61 significantly enhanced monocyte migration in the presence of Pcdh7, while DT-61 had no effect in the absence of Pcdh7. These results indicate that, although PP2A played a role in monocyte migration, its forced activation was insufficient to restore the impaired migration of *Pcdh7^−/−^* monocytes. Pcdh7 deficiency in monocytes resulted in no significant defects regarding PP2A, as its expression levels and activity were comparable between *Pcdh7^+/+^* and *Pcdh7^−/−^* monocytes [14]. Given this, the impaired activation of RhoA and Rac1 in *Pcdh7^−/−^* monocytes is not attributed to PP2A. It is plausible that an additional Pcdh7-mediated signaling pathway regulates small GTPase activation and monocyte migration. Further studies are required to clarify the mechanisms underlying the Pcdh7-mediated regulation of monocyte activity.

Taken together, we provide evidence of a novel role for Pcdh7 in monocyte motility. It is worth noting that the Gene Ontology annotations related to Pcdh7 include calcium-ion binding (GO:0005509), plasma membrane (GO:0005886), and cell adhesion (GO:0007155). Calcium ions play a critical role in cell migration by regulating various processes, including cytoskeletal reorganization, cell adhesion, focal adhesion, migrasome formation, and cell directionality [41,42,43]. This information suggests a potential role for Pcdh7 in regulating calcium ion activity and cell adhesion and coordinating cell migration. As impaired cell migration is a hallmark of many diseases, including autoimmune disease, inflammatory bowel diseases, neurological disorders, and cancer [44,45,46], a better understanding of the mechanisms underlying cell migration would be valuable for human medicine. Our findings suggest that Pcdh7 may serve as a potential new therapeutic target for conditions involving impaired cell migration.

## 4. Materials and Methods

### 4.1. In Vitro Cell Culture

BMMs from wild-type (*Pcdh7^+/+^*) and Pcdh7-deficient (*Pcdh7^−/−^*) mice were prepared as described in a previous publication [12]. In brief, whole bone marrow cells were extracted from the femurs and tibias of mice and incubated in 100 mm Petri dishes in α-MEM medium containing 10% fetal bovine serum and M-CSF (5 ng/mL) overnight. Non-adherent cells were collected and cultured for three days with M-CSF (60 ng/mL) to generate BMMs. AR-A014418 (Cat.# A3230) was purchased from Sigma (St. Louis, MO, USA). DT-016 (Cat.# S8774) was purchased from Shelleckchem (Houston, TX, USA).

### 4.2. Retrovirus Preparation and Transduction

Retroviral particles were prepared as described previously [13]. In brief, pMX-Pcdh7, pMX-caRhoA, pMX-caRac1, and empty pMX vectors were transfected into Plat-E packaging cells using PEImax (Polysciences, Warrington, PA, USA). After three days, the medium containing the retrovirus was harvested and passed through a syringe filter (0.45 μm pore diameter). BMMs were transduced with the retroviruses overnight with hexadimethrine bromide (8 μg/mL) in the presence of M-CSF (60 ng/mL). The infected cells were then selected by culturing for 2 days in the presence of puromycin (2 μg/mL) with M-CSF (60 ng/mL). Puromycin-resistant BMMs were used for the following experiments.

### 4.3. Migration Assay

BMMs were seeded into each well of the culture inserts (Ibidi™ LCC, Verona, WI, USA) at a density of 5 × 10^5^ cells/mL (100 µL per well). After overnight culture, a cell-free gap was created by removing the silicone insert. The cells were further cultured for 0, 12, and 24 h. Images were captured at the indicated time points, and the area of cell migration was measured using ImageJ software v1.54f (NIH ImageJ; NIH, Bethesda, MD, USA). Cell migration is expressed as a percentage of the total cell migration area.

### 4.4. Active GTPase and Western Blotting

The activities of small GTPase RhoA and Rac1 were measured using a RhoA/Rac1/Cdc42 Activation Assay Combo Biochem kit (Cat.# BK030, Cytoskeleton Inc. Denver, Co, USA) according to the manufacturer’s instructions. For Western blotting, whole lysates were collected using the RIPA lysis buffer (Cat.#9806S, Cell Signaling Technology, Beverly, MA, USA), and the protein quantity was measured. The proteins were subjected to electrophoretic separation and transferred to PVDF membranes. Blocker™ BSA (Thermo Scientific, Waltham, MA, USA) was used as the blocking buffer. The blots were then probed with the following antibodies: anti-RhoA (Cat.# ARH04, Cytoskeleton, Inc, Denver, CO, USA) and anti-Rac1 (Cat.# ARC03, Cytoskeleton, Inc, Denver, CO, USA).

### 4.5. Statistical Analysis

All the experiments were analyzed using one-way ANOVA or two-way ANOVA, followed by Tukey’s test, using Prism 10.0 (GraphPad Software Inc, San Diego, CA, USA). *p* < 0.05 was considered statistically significant.

## Data Availability

Data are contained within the article.
